# Circular RNA hsa_circ_0002483 promotes growth and invasion of lung adenocarcinoma by sponging miR-125a-3p

**DOI:** 10.1186/s12935-021-02241-y

**Published:** 2021-10-12

**Authors:** Jun Wan, Guanggui Ding, Min Zhou, Xiean Ling, Zhanpeng Rao

**Affiliations:** Department of Thoracic Surgery, Shenzhen People’s Hospital (The Second Clinical Medical College, Jinan University, The First Affiliated Hospital, Southern University of Science and Technology), Shenzhen, 518020 China

**Keywords:** hsa_circ_0002483, miR-125a-3p, Growth, Invasion, Lung adenocarcinoma

## Abstract

**Background:**

Increasing evidence indicates that the aberrant expression of circular RNAs (circRNAs) is involved in the pathogenesis and progression of lung adenocarcinoma (LUAC). However, the function and molecular mechanisms of hsa_circ_0002483 (circ_0002483) in LUAC remain unclear.

**Methods:**

The association between circ_0002483 expression and clinicopathological characteristics and prognosis in patients with LUAC was analyzed by fluorescence in situ hybridization. The functional experiments such as CCK-8, colony formation and Transwell assays and a subcutaneous tumor model were conducted to determine the role of circ_0002483 in LUAC cells. The specific binding between circ_0002483 and miR-125a-3p was validated by RNA immunoprecipitation, luciferase gene report and qRT-PCR assays. The effects of circ_0002483 on miR-125a-3p-mediated C-C motif chemokine ligand 4 (CCL4)-CCR5 axis were assessed by Western blot analysis.

**Results:**

We found that circ_0002483 was upregulated in LUAC tissue samples and associated with Tumor Node Metastasis (TNM) stage and poor survival in patients with LUAC. Knockdown of circ_0002483 inhibited proliferation, colony formation and invasion of A549 and PC9 cells in vitro, whereas overexpression of circ_0002483 harbored the opposite effects. Furthermore, circ_0002483 sponged miR-125a-3p and negatively regulated its expression. CCL4 was identified as a direct target of miR-125a-3p. The rescue experiments showed that miR-125a-3p mimics reversed the tumor-promoting effects of circ_0002483 by targeting CCL4-CCR5 axis in A549 and PC9 cells. In addition, the in vivo experiment further validated that knockdown of circ_0002483 repressed tumor growth.

**Conclusions:**

Our findings demonstrated that circ_0002483 could act as a sponge of miR-125a-3p to upregulate CCL4-CCR5 axis, contributing to the tumorigenesis of LUAC, and represent a potential therapeutic target for LUAC.

**Supplementary Information:**

The online version contains supplementary material available at 10.1186/s12935-021-02241-y.

## Introduction

Lung cancer is one of the most malignant tumors and its incidence and mortality are increasing rapidly, threatening to the public health and human life [1]. With the development of treatment methods, lung adenocarcinoma (LUAC) as a subclass of non-small cell lung cancer (NSCLC) has been well-treated, but the advanced cases still harbor a poor prognosis duo to its distant metastasis [2]. Accumulating data display that the aberrant expression of noncoding RNAs is associated with the prognosis and progression of LUAC [3–5]. Therefore, identification of cancer-related noncoding RNAs may provide potential biomarkers for the early detection of LUAC.

Circular RNA (circRNA) as a subclass of noncoding RNAs is characterized by covalently closed loop structures and RNA stability owing to resistance to RNase R [6]. Increasing data demonstrate that circRNAs are aberrantly expressed and associated with the prognosis and progression of LUAC [7–9]. Meanwhile, circRNAs act as oncogenic or suppressive factors in LUAC by interacting with RNA-binding protein or sponging miRNAs. For example, circXPO1 facilitates LUAC progression by interacting with IGF2BP1 [10] and circ_0001588 contributes to LUAC by sponging miR-524-3p/NACC1 signaling [11]. circ-CAMK2A, circ-ANXA7 and circ-AASDH promote LUAC metastasis by regulating miR-615-5p/fibronectin1, miR-331/LAD1 and miR-140-3p/E2F7 axis [12–14], while circ_0018414 and circ-MTO1 suppress LUAD proliferation by sponging miR-6807-3p and miR-17 [15, 16]. Until now, the functional of circ_0002483 in LUAD remains undocumented.

MicroRNAs (miRNAs) as another substyle of noncoding RNAs can target mRNAs by post-transcriptional levels and serve as promising biomarkers for early diagnosis and prognosis of LUAC [17]. Some studies show that miR-125a-3p and miR-125a-5p, downregulated in NSCLC, possess the inverse effects on migration and invasion of lung cancer cells [18], and downregulation of miR-125a-3p is associated with tumorigenesis and poor prognosis in patients with NSCLC [19]. Moreover, miR-125a-3p represses lung cancer growth and invasion by regulating the mouse double minute 2 homolog/p53 signaling [20], and NSCLC proliferation and migration by targeting metastasis-associated gene 1 [21]. miR-125a-3p is also sponged by lncRNA MALAT1 in hepatocellular carcinoma [22], circ-MAPK4 in gliomas, circ_0012919 in systemic lupus erythematous and circLMF1 in aortic smooth muscle cells [23–25].

In the present study, we identified a differentially-expressed hsa_circ_0002483 between LUAC and normal tissue samples. The tumor node metastasis (TNM) staging system for LUAD is an internationally accepted system used to determine the disease stage, guide management and determine prognosis. We found that the upregulation of circ_0002483 was associated with TNM stage and poor survival in patients with LUAC. Further in vitro and in vivo experiments verified that circ_0002483 promoted the growth and invasion of LUAC cells by sponging miR-125a-3p and upregulating CCL4-CCR5 axis, thereby providing a novel target for LUAC.

## Materials and methods

### Clinical samples

The tissue microarray (No. XT17-002) including 80 pair-matched LUAC samples was purchased from Shanghai Outdo Biotechnology (Shanghai, China). The clinical data of patients with LUAC as well as the expression of miR-125a-3p, miR-134-5p, miR-301a-5p, miR-222-5p, miR-501-5p, CCL4 and CCR5 were downloaded from TCGA dataset (http://xena.ucsc.edu/). The patients did not receive any chemotherapy, and the protocols were approved by the Ethics Committee of The Shenzhen People’s Hospital.

### Bioinformatic analysis

The circRNA profiling was used to identify the differentially-expressed circRNAs between LUAC and normal tissues and downloaded from the Gene Expression Omnibus (GEO) dataset (https://www.gcbi.com.cn/gclib/html/index).

### Fluorescence in situ hybridization (FISH)

The probe sequence for hsa_circ_0002483 (5′-GTATCTGTCATATTCTGTTGATAG AAGAAAAAAAAACGTGTCGCAGCCGTCAAGAGTGTCTAGGCAT-3′) and Biotin-labeled probe sequences for miR-125a-3p (5′-ACAGGTGAGGTTCTTGGGA GCCAAAAAAAACTGGACGCTAATAGCATAGACACTCTTAGA-3′) were used to analyze the expression of hsa_circ_0002483 (green fluorescent signal) and miR-125a-3p (red fluorescent signal) in LUAC tissue samples. The detailed description of FISH analysis was executed as previously reported [26].

### Cell culture and transfection

LUAC cell lines (A549 and PC9) used in these studies were provided by Cell bank of Shanghai Chinese Academy of Sciences (Shanghai, China), and cultured in DMEM (Gibco, Rockford, MD, USA) medium supplemented with 10% heat-inactivated FBS (Gibco, Rockford, MD, USA) in a humidified atmosphere containing 5% CO_2_ at 37 °C. Lentivirus mediated si-circ_0002483 (5′-AACAGAATATGACAGATACCTdTdT-3′), its negative control (si-NC), circ_0002483 overexpression plasmids, miR-125a-3p mimics and inhibitors were provided by GenePharma (Shanghai, China) and used for transfection into A549 and PC9 cells.

### Quantitative real-time (qRT-PCR)

RNA was extracted from A549 and PC9 cell lines using Trizol reagent (Invitrogen). One Step SYBR® PrimeScript™ PLUS RT-PCR Kit (Beijing, China) was used to detect the expression of circ_0002483, CCL4 and CCR5 in A549 and PC9 cells. TaqMan® MicroRNA Reverse Transcription Kit and TaqMan Universal Master Mix II (Thermo Fisher Scientific, Runcorn, UK) were used to examine miR-125a-3p levels. U6 or β-actin was used as an internal control. The data were quantified using 2^−∆∆CT^ equation in triplicate. The primer sequences used were shown in Additional file 1: Table S1.

### Western blot analysis

A549 and PC9 cell lines were harvested and protein was extracted using RIPA lysis. Primary antibodies against anti-CCL4 (ab25129, abcam), anti-CCR5 (ab65850, abcam) and anti-GAPDH (ab8245, abcam) were diluted (1:1000) and incubated overnight at 4 °C. After rinsing, the polyvinylidene fluoride (PVDF) membrane of the antibodies was transferred onto the system. Captured signal was quantified by Image Lab Software 3.0 (Bio-Rad), and GAPDH was used as an internal parameter.

### Cell proliferation assay

Cell proliferation was measured by a CCK-8 assay kit (Dojindo Corp, Japan). A total of 2000 cells were plated in each well of a 96-well plate. Then, on the indicated day, 10 µl of CCK-8 reagent was added directly to the culture medium. Then, the cells were incubated for 2.5 h at 37 °C, and the optional density was measured at 450 nm. These experiments were repeated three times.

### Colony formation assay

A549 and PC9 cells were trypsinized, and 1 × 10^3^ cells were plated in 6-well plates and incubated at 37 °C for 7 days. Colonies were dyed with dyeing solution containing 0.1% crystal violet and 20% methanol. Cell colonies were then counted and analyzed.

### Transwell invasion assay

The invasion assay was conducted by Transwell assay. The upper surfaces of Transwell filters were coated with Matrigel (BD, New Jersey, USA). Transfected cells (4 × 10^5^) in 200 µl of serum-free medium were added to the upper compartment of the chamber. A total of 500 µl of medium supplemented with 10% FBS was added into the lower chamber. The invaded cells were harvested after incubation for 48 h. The non-invaded cells on the upper side of the chamber were scraped off with a cotton swab. The cells were fixed in 4% paraformaldehyde and stained with a crystal violet solution. The cells were then counted and analyzed. 5-6 fields per chamber have been assessed for the assay.

### Actinomycin D and RNase R treatment

Transcription was prevented by the addition of 2 mg/ml Actinomycin D and DMSO (Sigma-Aldrich, St. Louis, MO, USA) was used as the control group. Total RNA was incubated for 30 min at 37 °Cwith 3 U/µg of RNase R (Epicentre Technologies, Madison, WI, USA).

### Dual-luciferase reporter assay

A549 and PC9 cells were seeded into 24-well plates, and PRL-TK-Luc report vectors containing WT or Mut 3’UTR of circ_0002483 and CCL4 were co-transfected with miR-125a-3p mimic or inhibitor into A549 and PC9 cell lines. After the transfection for 48 h, luciferase activities were detected with a dual-luciferase reporter system (Promega, Madison, WI).

### RNA immunoprecipitation

RNA immunoprecipitation (RIP) assay was conducted using a Magna RIP RNA-Binding Protein Immunoprecipitation Kit (Millipore, Billerica, MA) according to the manufacturer’s instructions.

### Animal experiments

Six-week-old female immune-deficient nude mice (BALB/c-nu) were injected subcutaneously with 5 × 10^7^ A549 cells stably transfected with si-circ_0002483 or si-NC. Mice were monitored daily and developed a subcutaneous tumor. The tumor volume was detected every other day by using a formula: volume = length × width^2^/2. This study was approved by Animal Ethics Committee of The Shenzhen People’s Hospital.

### Immunohistochemistry (IHC)

Ki-67 levels in xenograft tumor tissues were assessed using IHC assay. The detailed description of IHC was conducted as previously reported [4].

### Statistical analysis

Statistical analyses were performed by SPSS 20.0 (IBM, SPSS, Chicago, IL, USA) and GraphPad Prism. Student’s t-test or Chi-square test was used to estimate the statistical significance for comparisons of two groups. Pearson correlation analysis was used to analyze the correlations. Overall survival curve was drawn with the Kaplan-Meier and log-rank test. *P* < 0.05 was considered statistical significance.

## Results

### Upregulation of circ_0002483 was associated with poor survival in patients with LUAC

**Fig. 1:**
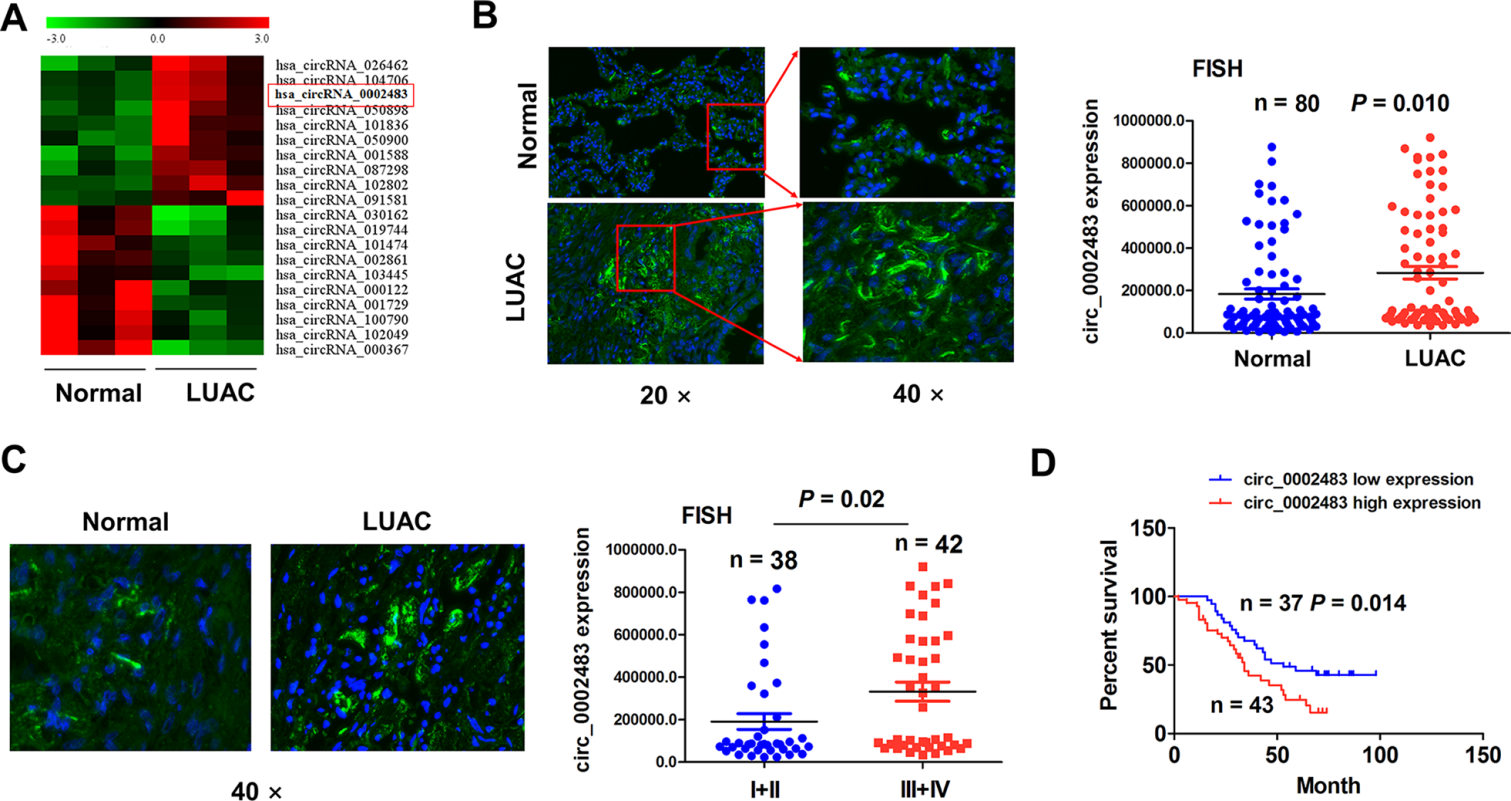
Upregulation of circ_0002483 was associated with poor survival in patients with LUAC. **A** Hierarchical clustering of differentially expressed circRNAs between LUAC and normal tissue samples. **B** FISH analysis of the expression levels of circ_0002483 in 80 pair-matched LUAC tissue samples. **C** FISH analysis of the expression levels of circ_0002483 in LUAC with stage I + II and stage III + IV. **D** Kaplan–Meier analysis of the association of circ_0002483 expression with overall survival in patients with LUAC

The GEO dataset (GSE101684) was used to screen the differentially-expressed circRNAs between LUAC and non-cancerous tissue samples and top 10 upregulated or downregulated circRNAs were identified according to the FC > 2 and *P* < 0.01, of which hsa_circ_0002483 harbored a remarkable elevation in LUAC (Fig. 1A). Then, FISH analysis validated that circ_0002483 expression levels were increased in pair-matched LUAC tissue samples as compared with the non-cancerous tissues (Fig. 1B; n = 80, *P* = 0.01). The similar result was shown in LUAC patients with stage III–IV (n = 42) as compared with those with stage I–II (n = 38) (Fig. 1C; *P* = 0.02).

In addition, we analyzed the association of circ_002483 with the clinicopathological characteristics in patients with LUAC and found that elevated expression of circ_002483 was related with TNM stage (Table 1; *P* = 0.048) rather than other parameters (*P* > 0.05) in LUAC. On the basis of the cutoff value of circ_002483, we divided the cases into circ_002483-high (n = 43) and circ_002483-low groups (n = 37). Kaplan-Meier analysis uncovered that the patients with circ_002483-high group possessed a poorer survival as compared with those with circ_002483-low expression (Fig. 1D; *P* = 0.014). Univariate and Multivariate analysis unveiled that TNM stage and pathological stage rather than circ_002483 expression are independent prognostic factors of poor survival in patients with LUAC (Additional file 1: Tables S2).

**Table 1:** The association of circ_0002483 expression with clinicopathological characteristics in patients with LUAC

Variables	Cases (n)	circ_0002483		*P* value
		High	Low	
Total	80	43	37	
Age (years)				
≥ 60	40	24	16	
< 60	40	19	21	0.265
Sex				
Male	41	22	19	
Female	39	21	18	0.987
Pathologic stage				
I–II	41	24	17	
III–IV	39	19	20	0.382
Tumor size (cm)				
≥ 3	57	33	24	
< 3	23	10	13	0.245
TNM staging				
I–II	38	16	22	
III–IV	42	27	15	0.048
Lymph node metastasis	l			
Negative	41	21	20	
Positive	39	22	17	0.644

## Identification of a novel circ_0002483 and its cellular localization

**Fig. 2:**
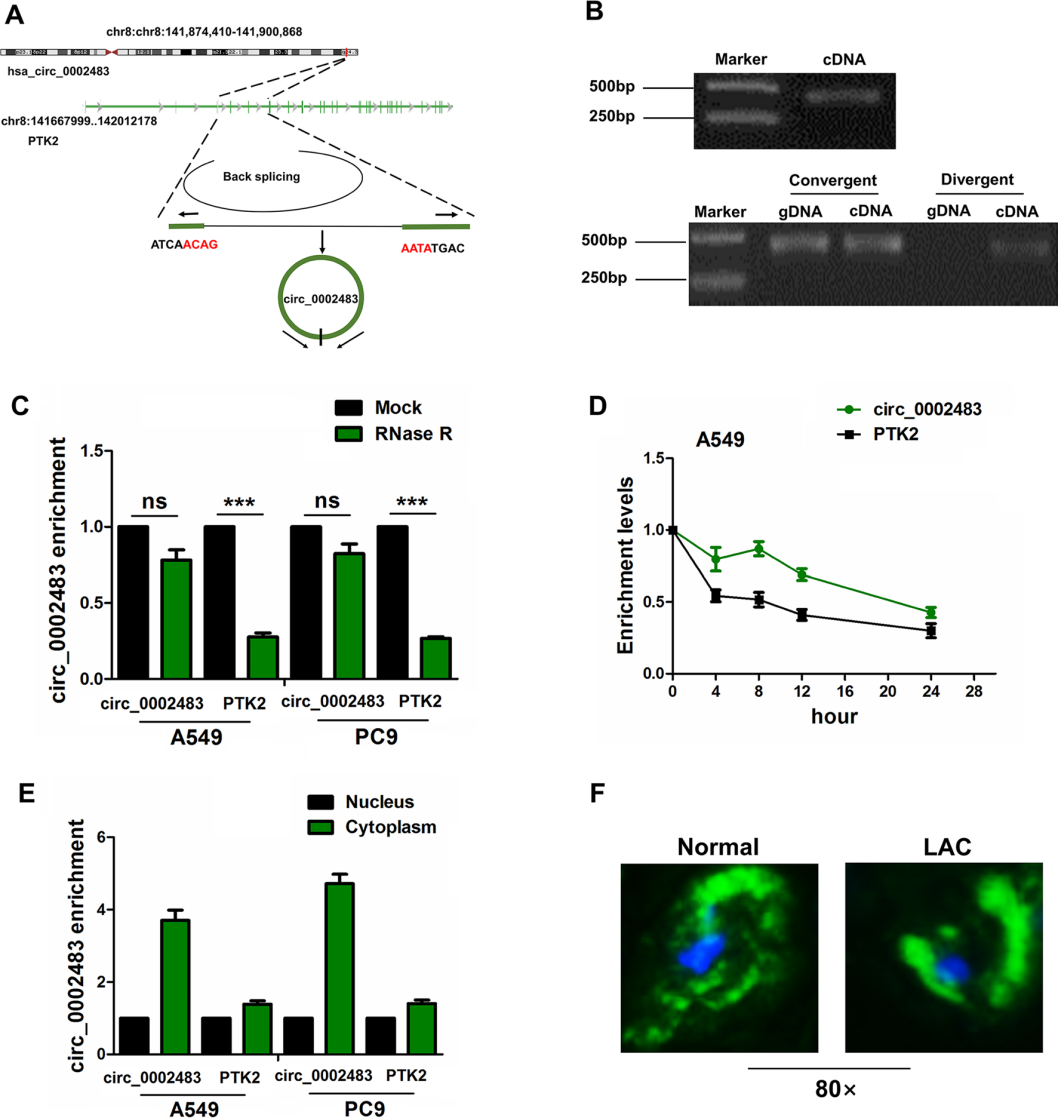
Identification of a novel circ_0002483 in LUAC cells. **A** The genomic loci of circ_0002483. **B** PCR product of circ_0002483 in agarose gel electrophoresis and convergent and divergent primers for confirmation of the closed loop structure. **C** qRT-PCR analysis of circ_0002483 and PTK2 expression levels after treatment with RNase R in A549 and PC9 cells. **D** qRT-PCR analysis of the stability of circ_0002483 and PTK2 after treatment with Actinomycin D in A549 cells. **E**, **F** qRT-PCR and FISH analysis of the location of circ_0002483 in LUAC cells and tissues. Data shown are the mean ± SEM of three experiments. ***P < 0.001

According to the annotation of circRNAs by Circular RNA Interactome (https://circinteractome.nia.nih.gov/index.html), we found that hsa_circ_0002483 (chr8:141874410-141900868) is originated from exon 2, 6 regions within protein tyrosine kinase 2 (PTK2) locus, and the spliced sequence is about 482 bp (Fig. 2A). The back-spliced junction of circ_0002483 was amplified by divergent primer and circ_0002483 could be amplified from only cDNA but not gDNA in A549 cells (Fig. 2B). Relative to linear PTK2, circ_0002483 produced a resistance to RNase R treatment in A549 and PC9 cell lines, and circ_0002483 harbored a loop structure in LUAC cells (Fig. 2C). After A549 cells were treated by Actinomycin D, qRT-PCR displayed that circ_0002483 was more stable than PTK2 (Fig. 2D). qRT-PCR and FISH analysis revealed that circ_0002483 was mainly localized in the cytoplasm of LUAC cells and tissues (Fig. 2E, F).

### Circ_0002483 facilitated proliferation, colony formation and invasion in vitro

**Fig. 3:**
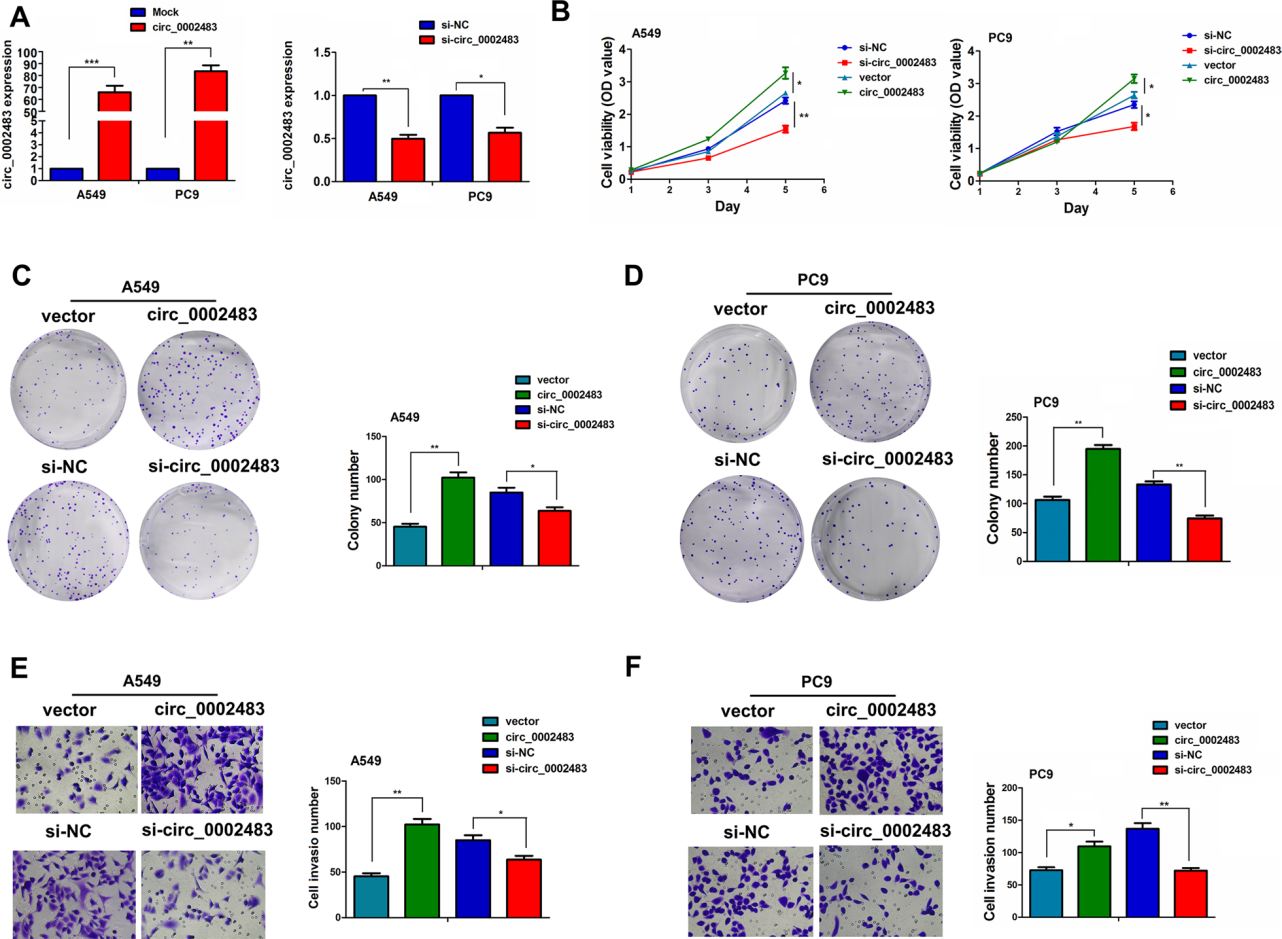
Circ_0002483 promoted proliferation, colony formation and invasion in vitro. **A** qRT-PCR analysis of the expression levels of circ_0002483 after the transfection with circ_0002483 overexpression vectors or si-circ_0002483 in A549 and PC9 cells. **B** CCK-8 analysis of the cell proliferation viability after transfection with circ_0002483 overexpression vectors or si-circ_0002483 in A549 and PC9 cells. **C**, **D** Colony formation analysis of cell colony number after transfection with circ_0002483 overexpression vectors or si-circ_0002483 in A549 and PC9 cells. **E**, **F** Transwell analysis of cell invasion capabilities after transfection with circ_0002483 overexpression vectors or si-circ_0002483 in A549 and PC9 cells. Data shown are the mean ± SEM of six experiments. *P < 0.05, **P < 0.01, ***P < 0.001

Upregulation of circ_0002483 in LUAC suggested that it might be an oncogenic factor. To test this hypothesis, we evaluated the function of circ_0002483 in A549 and PC9 cells and constructed the overexpression vectors and the siRNA against circ_0002483. As indicated in Fig. 3A, the overexpression vectors and the siRNA of circ_0002483 could markedly increase and decrease the expression of circ_0002483 in A549 and PC9 cell lines, respectively. CCK-8 assay was conducted to chart the growth curve which indicated that upregulation of circ_0002483 obviously increased the proliferation viability of A549 and PC9 cells, while downregulation of circ_0002483 repressed cell growth (Fig. 3B). Colony formation assay further verified that the cell colony number of A549 and PC9 was remarkably elevated by upregulation of circ_0002483 and reduced by downregulation of circ_0002483 (Fig. 3C, D). In addition, the effects of circ_0002483 on LUAC cell invasion were estimated by Transwell assay, which indicated that overexpression of circ_0002483 increased invasive capabilities of A549 and PC9 cells, while knockdown of circ_0002483 harbored the opposite effects (Fig. 3E, F).

### Circ_0002483 was negatively associated with miR-125a-3p expression in LUAC tissue samples

**Fig. 4:**
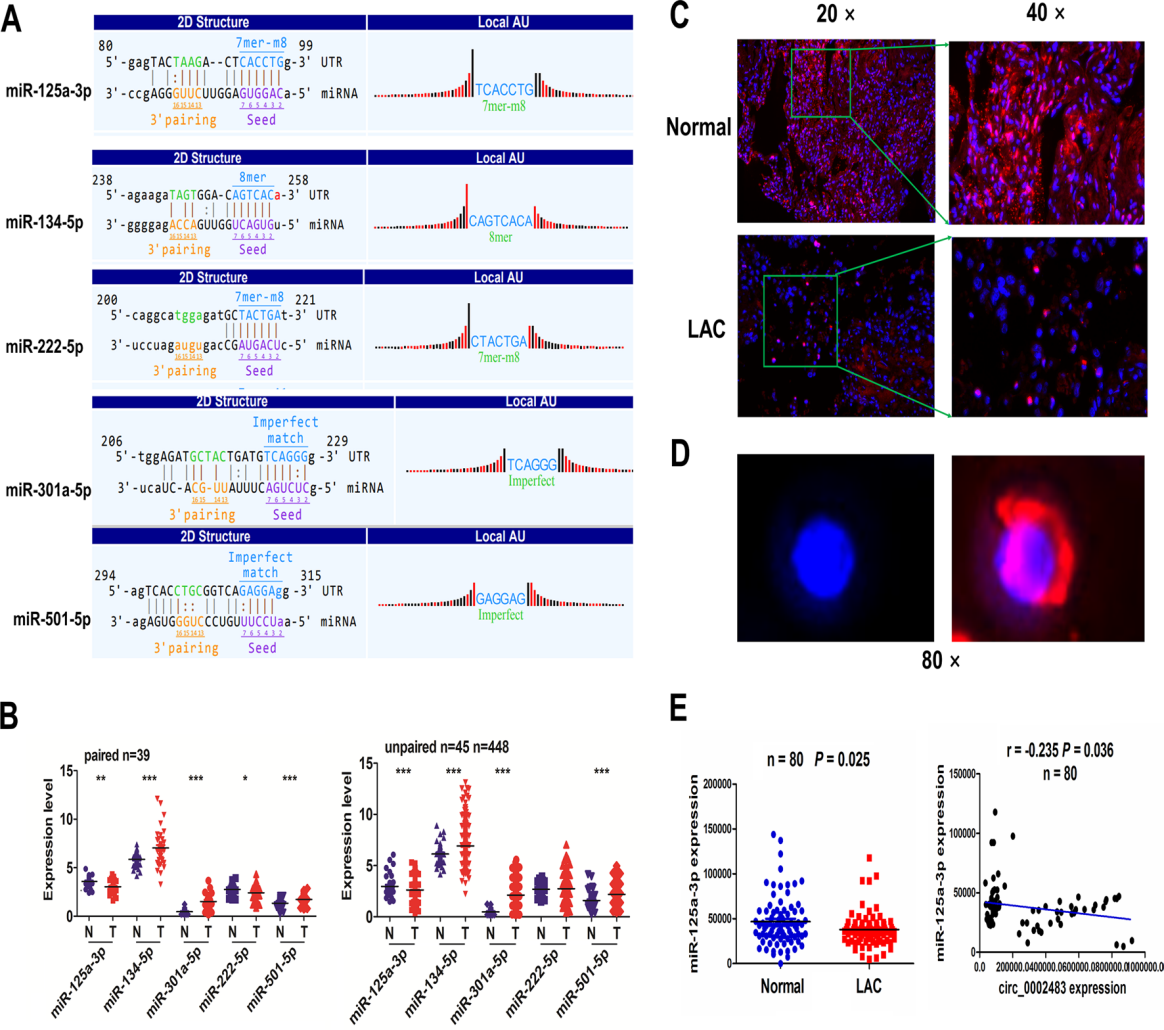
Circ_0002483 harbored a negative correlation with miR-125a-3p expression in LUAC tissue samples. **A** The binding sites between circ_0002483 and multiple miRNAs. **B** TCGA analysis of the expression levels of 5 miRNAs in paired and unpaired LUAC tissue samples. **C**–**E** FISH analysis of the expression levels and cellular location of miR-125a-3p, and Pearson correlation analysis of the correlation of circ_0002483 with miR-125a-3p expression in LUAC tissue samples (n = 80)

To demonstrate the underlying mechanisms of circ_0002483 in LUAC cells, we identified the binding sites of 5 miRNAs with circ_0002483 3’UTR by Circular RNA profiling and Interactome (Fig. 4A). TCGA cohort showed that, relative to the other 4 miRNAs, only miR-125a-3p possessed a decreased expression in paired (n = 39) and unpaired LUAC tissue samples (n = 448, Fig. 4B). The downregulation and cytoplastic location of miR-125a-3p in pair-matched LUAC tissues were further confirmed by FISH analysis (n = 80, *P* = 0.025; Fig. 4C–E). Pearson correlation analysis demonstrated that circ_0002483 harbored a negative correlation with miR-125a-3p expression in LUAC tissue samples (*P* = 0.036; Fig. 4E).

Then, we analyzed the association of miR-125a-3p with clinicopathological characteristics in patients with LUAC (Additional file 1: Table S3) and found that miR-125a-3p expression was related with gender (*P* = 0.012) and lymph node metastasis (*P* = 0.013) in patients with LUAC. However, the patients with miR-125a-3p-low expression showed no difference in poor survival and tumor recurrence as compared with those with miR-378a-3p-high expression (Additional file 2: Figure S1).

### Circ_0002483 acted as a sponge of miR-125a-3p

**Fig. 5:**
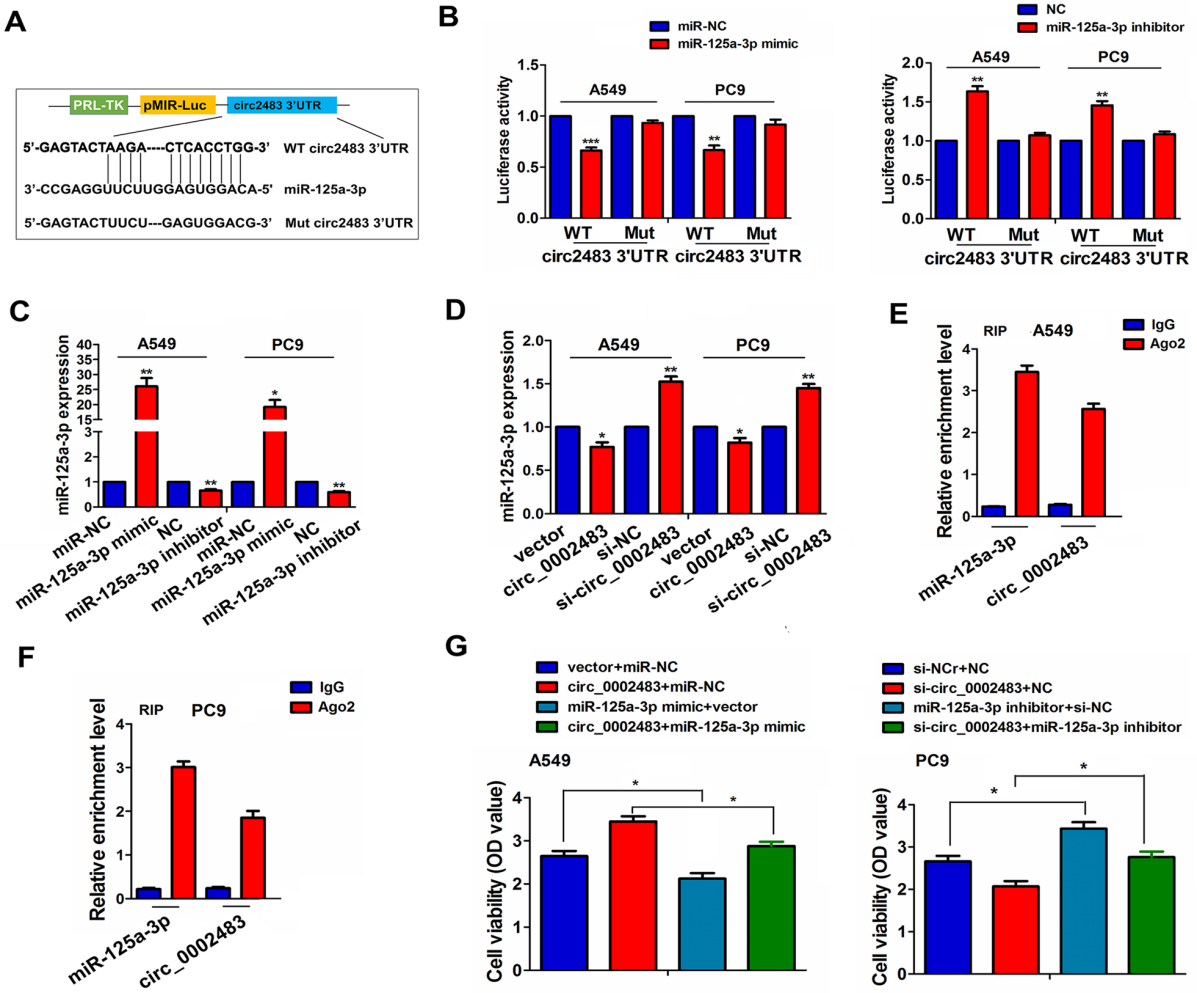
Circ_0002483 acted as a sponge of miR-125a-3p in LUAC cells. **A** Schematic representation of the binding sites of miR-125a-3p with WT or Mut circ_0002483 3′UTR. **B** Estimation of luciferase activities of WT or Mut circ_0002483 3′UTR after co-treatment with miR-125a-3p mimics or inhibitor and WT or Mut circ_0002483 3’UTR reporter vectors in A549 and PC9 cells. **C** qRT-PCR analysis of the expression levels of miR-125a-3p after transfection with its mimics and inhibitor in A549 and PC9 cells. **D** qRT-PCR analysis of the expression levels of miR-125a-3p after transfection with circ_0002483 overexpression vectors or si-circ_0002483 in A549 and PC9 cells. **E**, **F** RIP analysis of the enrichment levels of circ_0002483 and miR-125a-3p pulled down from Ago2 protein in A549 and PC9 cells. **G** CCK-8 analysis of cell proliferation viability after co-transfection with circ_0002483 overexpression vectors and miR-378a-3p mimics in A549 cells or si-circ_0002483 and miR-125a-3p inhibitor in PC9 cells. Data shown are the mean ± SEM of three experiments. *P < 0.05, **P < 0.01, ***P < 0.001

The specific binding sites of miR-125a-3p with WT or Mut circ_0002483 3′UTR were demonstrated in Fig. 5A. To determine the binding of circ_0002483 with miR-125a-3p, we found that miR-125a-3p mimics decreased the luciferase activities of WT circ_0002483 3′UTR in A549 and PC9 cells, while miR-125a-3p inhibitor increased its luciferase activities (Fig. 5B). But, miR-125a-3p exhibited no effects on those of Mut circ_0002483 3′UTR. Further qRT-PCR analysis indicated that upregulation of circ_0002483 reduced the expression of miR-125a-3p, and downregulation of circ_0002483 increased its expression (Fig. 5C, D), whereas miR-125a-3p exhibited no effects on circ_0002483 expression in A549 and PC9 cells (Additional file 3: Figure S2). Moreover, RIP assay was used to confirm the binding of circ_0002483 or miR-125a-3p with Ago2 protein in A549 and PC9 cells. We found that the endogenous expression of circ_0002483 and miR-125a-3p pulled down from Ago2 protein in A549 and PC9 cells was markedly increased as compared with the IgG control group (Fig. 5E, F). After co-transfection with circ_0002483 and miR-125a-3p mimics or si-circ_0002483 and miR-125a-3p inhibitors into A549 and PC9 cells for 5 days, we found that miR-125a-3p suppressed the proliferation viability and attenuated circ_0002483-caused cell proliferation, while miR-125a-3p inhibitors displayed the opposite effects (Fig. 5G).

### MiR-125a-3p reversed circ_0002483-caused upregulation of CCL4-CCR5 axis

**Fig. 6:**
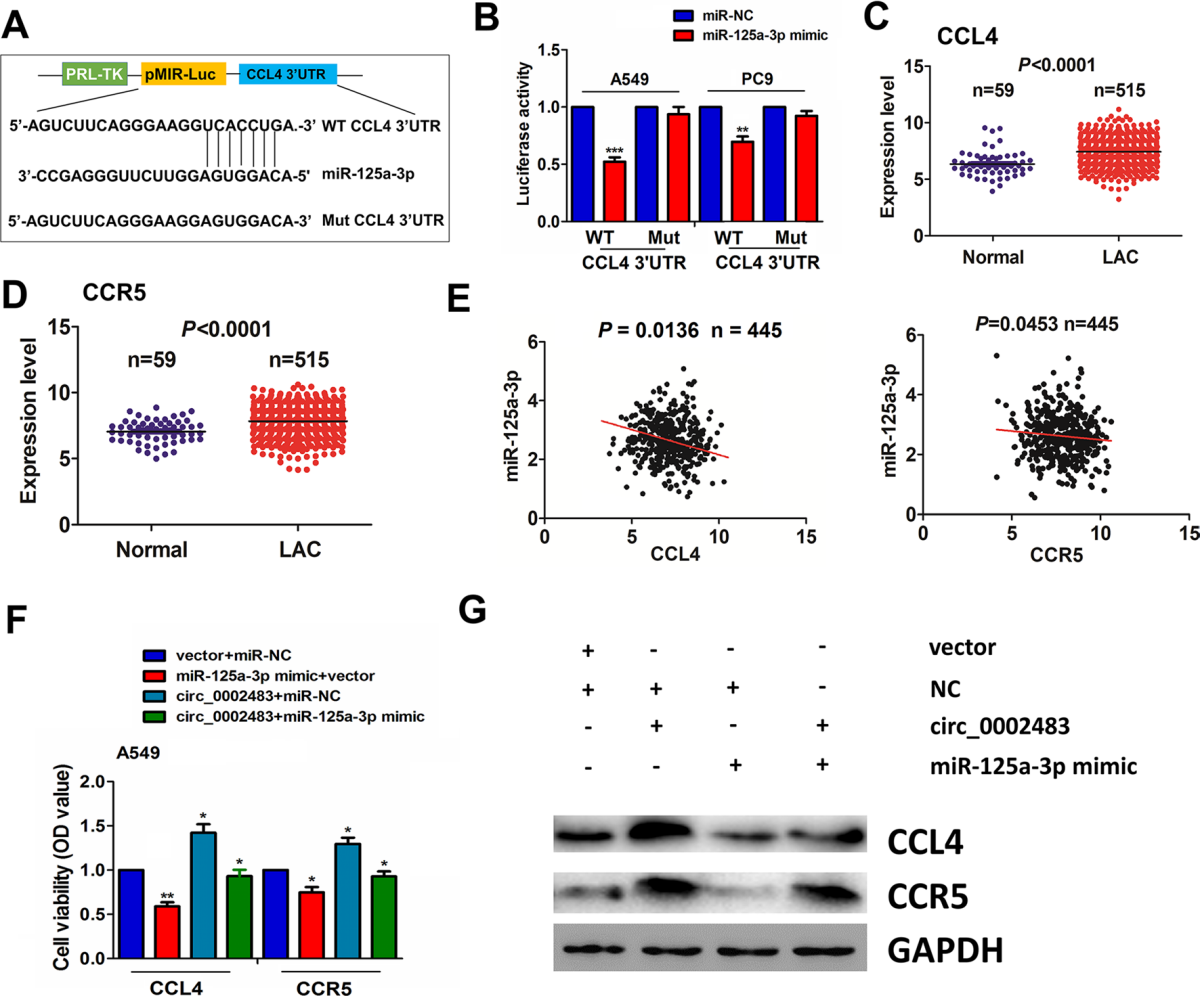
miR-125a-3p reversed circ_0002483-caused upregulation of CCL4-CCR5 axis. **A** Schematic representation of the binding sites of miR-125a-3p with WT or Mut CCL4 3′UTR. **B** Estimation of luciferase activities of WT or Mut CCL4 3′UTR after co-treatment with miR-125a-3p mimics and WT or Mut CCL4 3’UTR reporter vectors in A549 and PC9 cells. **C**, **D** TCGA analysis of the expression levels of CCL4 and CCR5 in LUAC tissue samples (n = 515). **E** Pearson correlation analysis of the correlation of miR-125a-3p with CCL4 and CCR5 expression in LUAC tissue samples. **F**, **G** qRT-PCR and Western blot analysis of the expression levels of CCL4 and CCR5 after co-transfection with circ_0002483 overexpression vectors and miR-125a-3p mimics in A549 cells. Data shown are the mean ± SEM of three experiments. *P < 0.05, **P < 0.01, ***P < 0.001

The targets of miR-125a-3p were identified by Targetscan7.1 and mirPathv.3, which indicated that C-C motif chemokine ligand 4 (CCL4) may be a direct target of miR-125a-3p. CCL4 can bind with its receptor CCR5, leading to tumorigenesis and progression. The binding sites of miR-125a-3p with 3′UTR of CCL4 were demonstrated in Fig. 6A. To determine the binding of miR-125a-3p with 3′UTR of CCL4, we co-transfected A549 and PC9 cells with WT or Mut CCL4 3′UTR reporter vectors and miR-125a-3p mimics, and found that miR-125a-3p mimics decreased the luciferase activities of WT CCL4 3’UTR in A549 and PC9 cells, however, miR-125a-3p exhibited no effects on those of Mut CCL4 3’UTR (Fig. 6B). TCGA cohort indicated that CCL4 and CCR5 were upregulated in 515 LUAC tissue samples (Fig. 6C, D; *P* < 0.0001) and miR-125a-3p harbored a negative correlation with both of them in LUAC (Fig. 6E; *P* < 0.05). In addition, we found that high expression of CCL4 harbored no association with clinicopathological features in patients with LUAC with (Additional file 1: Table S4), and the patients with CCL4-high expression possessed no difference in poor survival and tumor recurrence relative to those with CCL4-low expression (Additional file 4: Figure S3). Moreover, qRT-PCR and Western blot analyses demonstrated that miR-125a-3p inhibited the expression of CCL4 and CCR5 and reversed circ_0002483-caused upregulation of CCL4-CCR5 axis in A549 cells (Fig. 6F, G).

### Knockdown of circ_0002483 repressed cell growth in vivo

**Fig. 7:**
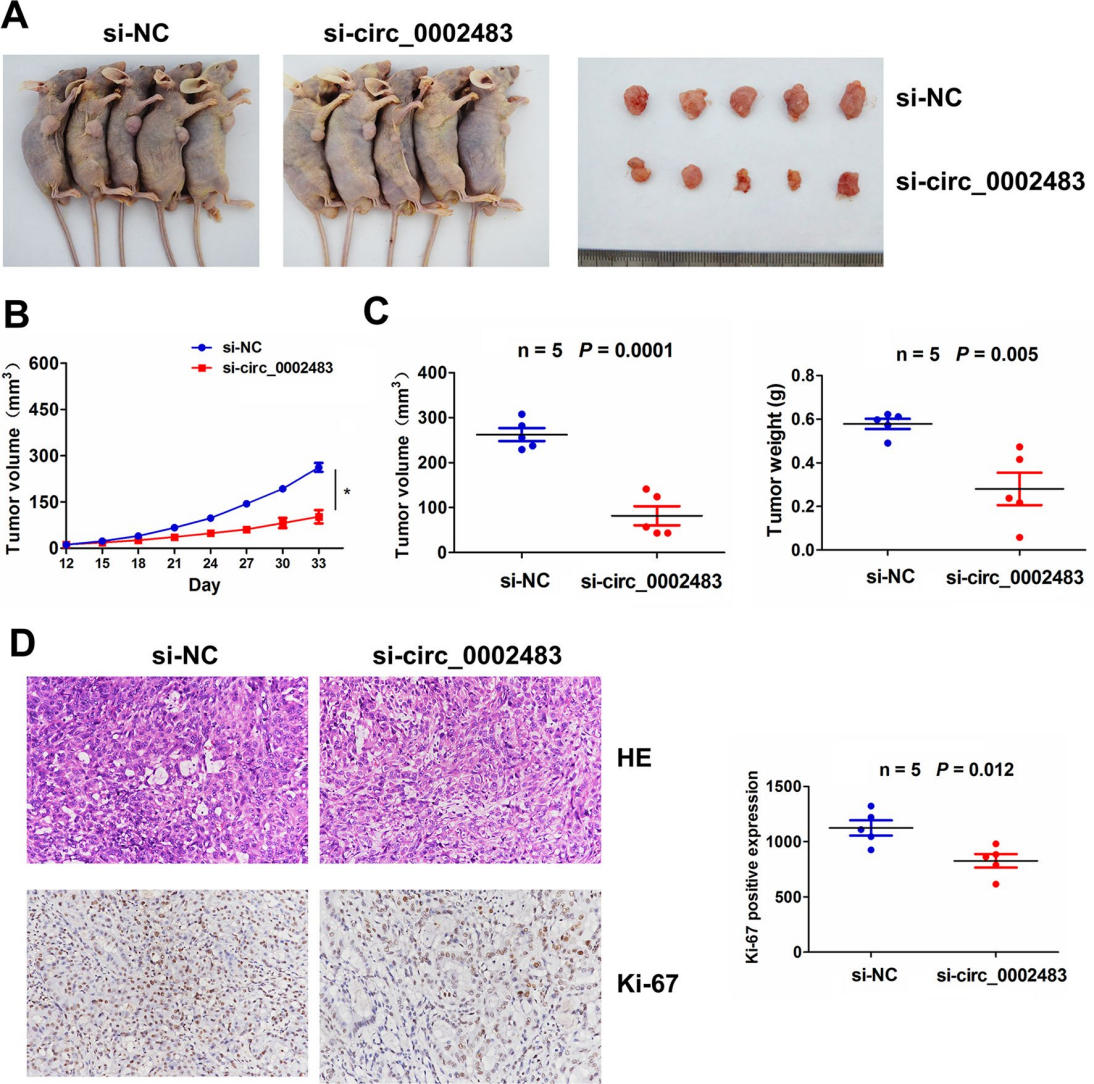
Knockdown of circ_0002483 repressed LUAC tumor growth. **A** Representative photographs of the subcutaneous xenograft tumors and assessment of the tumor growth curve after treatment with si-circ_0002483 or si-NC transfected A549 cells. **B**, **C** Comparison of tumor volume and weight between si-circ_0002483 and si-NC groups. **D** HE and IHC analysis of the Ki-67 expression levels between si-circ_0002483 and si-NC groups

To ascertain the effects of circ_0002483 on LUAC tumor growth in vivo, stably transfected A549 cells infected with si-NC or si-circ_0002484 were constructed and subcutaneously injected into 6-week old female nude mice (Fig. 7A). Moreover, the tumor volume and weight in the si-circ_0002384 group were smaller and lighter than the si-NC group (Fig. 7B, C). Hematoxylin and eosin (HE) staining and IHC of Ki-67 indicated that knockdown of circ_0002483 inhibited cell proliferation as compared with the si-NC group (Fig. 7D).

## Discussion

A sea of studies have shown that the aberrant expression of circRNA is related with the prognosis, pathogenesis and progression in multiple cancers including LUAC [26–28]. hsa_circ_0000792 and circ_0013958 are identified as potential biomarkers of LUAD [7, 9] and hsa_circ_0000190 is linked to tumor progression and poor prognosis in advanced lung cancer [8]. The tissue and plasmid levels of hsa_circ_0003221 (circPTK2) are correlated with poor differentiation and lymph node metastasis in bladder cancer [29]. Herein, we identified a novel differentially-expressed hsa_circ_0002483 (circPTK2) in LUAD tissue samples and found that, elevated expression of circ_0002483 was associated with TNM stage and poor survival in patients with LUAD and might provide a potential prognostic factor for LUAD.

Previous studies indicated that circRNAs have double-effects in LUAD progression. For example, circCSNK1G3 and circ-SOX4 facilitate growth and metastasis of LUAC by sponging miR-143-3p and miR-1270 [30, 31], whereas circCRIM1 and circ-000881 repress LUAC by sponging miR-182 and miR-665 [32, 33]. Moreover, circ_0008305 (circPTK2) is shown to inhibit the metastasis of NSCLC [34]. Herein, we found a novel circ_0002483 (circPTK2) and confirmed that knockdown of circ_0002483 suppressed growth and invasion of LUAD cells, while ectopic expression of circ_0002483 exhibited the tumor-promoting effects. Our results indicated that circ_0002483 might be an oncogenic factor in LUAC.

Increasing studies have indicated that circRNAs act as miRNA sponges to regulate LUAD progression [30–33]. Herein, we found that circ_0002483 could bind with Ago2-miR-125a-3p complex and negatively regulate miR-125a-3p expression in LUAC cells. Previous studies showed that miR-125a-3p can be also sponged by circ-MAPK4 and circARFGEF1 to regulate cell proliferation and invasion in glioma and LUAC [23, 35]. These studies suggested that, circ_0002483 might act as a sponge of miR-125a-3p to promote LUAC progression.

It has been shown that miR-125a-3p is downregulated and inhibits cell growth and invasion of LUAC [18–21]. In accordance, we found that miR-125a-3p expression was decreased in LUAC and associated with lymph node metastasis in patients with LUAC. miR-125a-3p harbored a negative correlation with circ_0002483 expression, repressed cell proliferation and reversed circ_0002483-induced tumor-promoting effects. In addition, CCL4-CCR5 axis contributes to breast cancer metastasis [36] and targeting CCR5 inhibits colorectal cancer liver metastasis [37]. We herein identified that CCL4 was upregulated in LUAC tissues and regarded as a direct target of miR-125a-3p. miR-125a-3p downregulated CCL4-CCR5 axis and reversed circ_0002483-caused upregulation of this axis. Our results suggested that circ_0002483 might act as a sponge of miR-125a-3p to upregulate CCL4-CCR5 axis, contributing to the tumorigenesis of LUAC.

Taken together, elevated expression of circ_0002483 is associated with TNM stage and poor survival in patients with LUAC. circ_0002483 facilitates the tumorigenesis and invasion of LUAC by sponging miR-125a-3p and upregulating CCL4-CCR5 axis. Our findings might offer a potential therapeutic biomarker for LUAC.

## Supplementary Information

**Additional file 1: Table S1.** The list of primer sequences. **Table S2. **Cox regression analysis of circ_0002483 expressionas a survival predictor in LUAC. **Table S3.** The association of miR-125a-3pexpression with clinicopathological characteristics in patients with LUAC. **Table S4.** The association ofCCL4 expression with clinicopathological characteristics in patients with LUAC

**Additional file 2: Figure S1. **Kaplan-Meier analysis of the association of miR-125a-3pexpression with overall survival in patients with LUAC.

**Additional file 3: Figure S2. **qRT-PCR analysis of the expression levels ofcirc_0002483 after transfection with miR-125a-3p mimics or inhibitors in A549and PC9 cells.

**Additional file 4: Figure S3. **Kaplan-Meier analysis of the association of CCL4expression with overall survival in patients with LUAC.

## Data Availability

The datasets used during the current study are available from the corresponding author on reasonable request.
