# Supplementary material for: Circular RNA hsa_circ_0002483 promotes growth and invasion of lung adenocarcinoma by sponging miR-125a-3p
Source: Cancer Cell Int. 2021 Oct 12;21:533. doi: 10.1186/s12935-021-02241-y (PMC8513360; doi:10.1186/s12935-021-02241-y)
Supplement: Supplementary file 1 — Additional file 1: Table S1. The list of primer sequences. Table S2. Cox regression analysis of circ_0002483 expressionas a survival predictor in LUAC. Table S3. The association of miR-125a-3pexpression with clinicopathological characteristics in patients with LUAC. Table S4. The association ofCCL4 expression with clinicopathological characteristics in patients with LUAC [file 12935_2021_2241_MOESM1_ESM.docx]

**Additional Tables**

**Table S1.** The list of primer sequences

| Markers | Sense (5’-3’) | Antisense (5’-3’) |
| --- | --- | --- |
| circ_0002483 | TTCGTTATTTGCCAAAAGGATT | TGTGATTCAAGTTGGGGTCA |
| β-actin | GCGTGACATTAAGGAGAAGC | CCACGTCACACTTCATGATGG |
| miR-125a-3p | ACAGGUGAGGUUCUUGGGAGCC | GGCUCCCAAGAACCUCACCUGU |
| U6 | CAGCACATATACTAAAATTGGAACG | ACGAATTTGCGTGTCATCC |
| CCL4 | CCGTGTTATTGTATTAGGTG | GAATCAAATGTGTTATCCATGT |
| CCR5 | TTCTGGGCTCCCTACAACATT | TTGGTCCAACCTGTTAGAGCTA |

**Table S2.** Cox regression analysis of circ_0002483 expression as a survival predictor in LUAC

| Variables | Univariate Cox regression analysis | |  | Multivariate Cox regression analysis | |
| --- | --- | --- | --- | --- | --- |
|  | RR (95% CI) | *P* value |  | RR (95% CI) | *P* value |
| ***Age (years)*** |  |  |  |  |  |
| ≥60 vs. <60 | 1.415 (0.805 to 2.486) | 0.228 |  |  |  |
| ***Sex*** |  |  |  |  |  |
| Mail vs. Female | 1.232 (0.702 to 2.161) | 0.467 |  |  |  |
| ***Pathological stage*** |  |  |  |  |  |
| III-IV vs. I-II | 2.261 (1.268 to 4.029) | 0.006 |  | 2.090 (1.157 to 3.769) | 0.014 |
| ***Tumor size (cm)*** |  |  |  |  |  |
| ≥3 vs. <3 | 1.054 (0.574 to 1.937) | 0.864 |  |  |  |
| ***TNM staging*** |  |  |  |  |  |
| III-IV vs. I-II | 3.167 (1.748 to 5.740) | <0.0001 |  | 2.471 (1.299 to 4.704) | 0.006 |
| ***Lymph node metastasis*** |  |  |  |  |  |
| Positive vs. Negative | 2.787 (1.547 to 5.020) | 0.001 |  | 1.830 (0.971 to 3.451) | 0.062 |
| ***circ_0002483 level*** |  |  |  |  |  |
| High VS. Low | 0.988 (0.562 to 1.735) | 0.966 |  | 0.927 (0.521 to 1.651) | 0.798 |

NA: not analyzed

**Table S3** The association of miR-125a-3p expression with clinicopathological characteristics in patients with LUAC

| Variables | Cases  (n) | miR-125a-3p | | *P* value |
| --- | --- | --- | --- | --- |
|  |  | High | Low |  |
| Total | 419 | 365 | 54 |  |
| ***Age (years)*** |  |  |  |  |
| ≥60 | 302 | 264 | 38 |  |
| <60 | 117 | 101 | 16 | 0.748 |
| ***Gender*** |  |  |  |  |
| Male | 194 | 160 | 34 |  |
| Female | 225 | 205 | 20 | 0.012 |
| ***Pathological stage*** |  |  |  |  |
| Ⅰ/Ⅱ | 332 | 288 | 44 |  |
| Ⅲ/Ⅳ | 87 | 77 | 10 | 0.723 |
| ***Tumor size (cm)*** |  |  |  |  |
| ≥ 3 | 363 | 319 | 44 |  |
| < 3 | 56 | 46 | 10 | 0.281 |
| ***Lymph node metastasis*** |  |  |  |  |
| Negative | 277 | 233 | 44 |  |
| Positive | 142 | 132 | 10 | 0.013 |
| ***TNM stage*** |  |  |  |  |
| Ⅰ/Ⅱ | 261 | 225 | 36 |  |
| Ⅲ/Ⅳ | 158 | 140 | 18 | 0.548 |

**Table S4** The association of CCL4 expression with clinicopathological characteristics in patients with LUAC

| Variables | Cases  (n) | CCL4 | | *P* value |
| --- | --- | --- | --- | --- |
|  |  | High | Low |  |
| Total | 481 | 257 | 224 |  |
| ***Age (years)*** |  |  |  |  |
| ≥60 | 350 | 189 | 161 |  |
| <60 | 131 | 68 | 63 | 0.683 |
| ***Gender*** |  |  |  |  |
| Male | 220 | 113 | 107 |  |
| Female | 261 | 144 | 117 | 0.411 |
| ***Pathological stage*** |  |  |  |  |
| Ⅰ/Ⅱ | 378 | 207 | 171 |  |
| Ⅲ/Ⅳ | 103 | 50 | 53 | 0.268 |
| ***Tumor size (cm)*** |  |  |  |  |
| ≥ 3 | 417 | 222 | 195 |  |
| < 3 | 64 | 35 | 29 | 0.893 |
| ***Lymph node metastasis*** |  |  |  |  |
| Negative | 312 | 176 | 136 |  |
| Positive | 169 | 81 | 88 | 0.085 |
| ***TNM stage*** |  |  |  |  |
| Ⅰ/Ⅱ | 318 | 167 | 151 |  |
| Ⅲ/Ⅳ | 163 | 90 | 73 | 0.629 |
