# Supplementary figures and images for: Circular RNA hsa_circ_0002483 promotes growth and invasion of lung adenocarcinoma by sponging miR-125a-3p
Source: Cancer Cell Int. 2021 Oct 12;21:533. doi: 10.1186/s12935-021-02241-y (PMC8513360; doi:10.1186/s12935-021-02241-y)

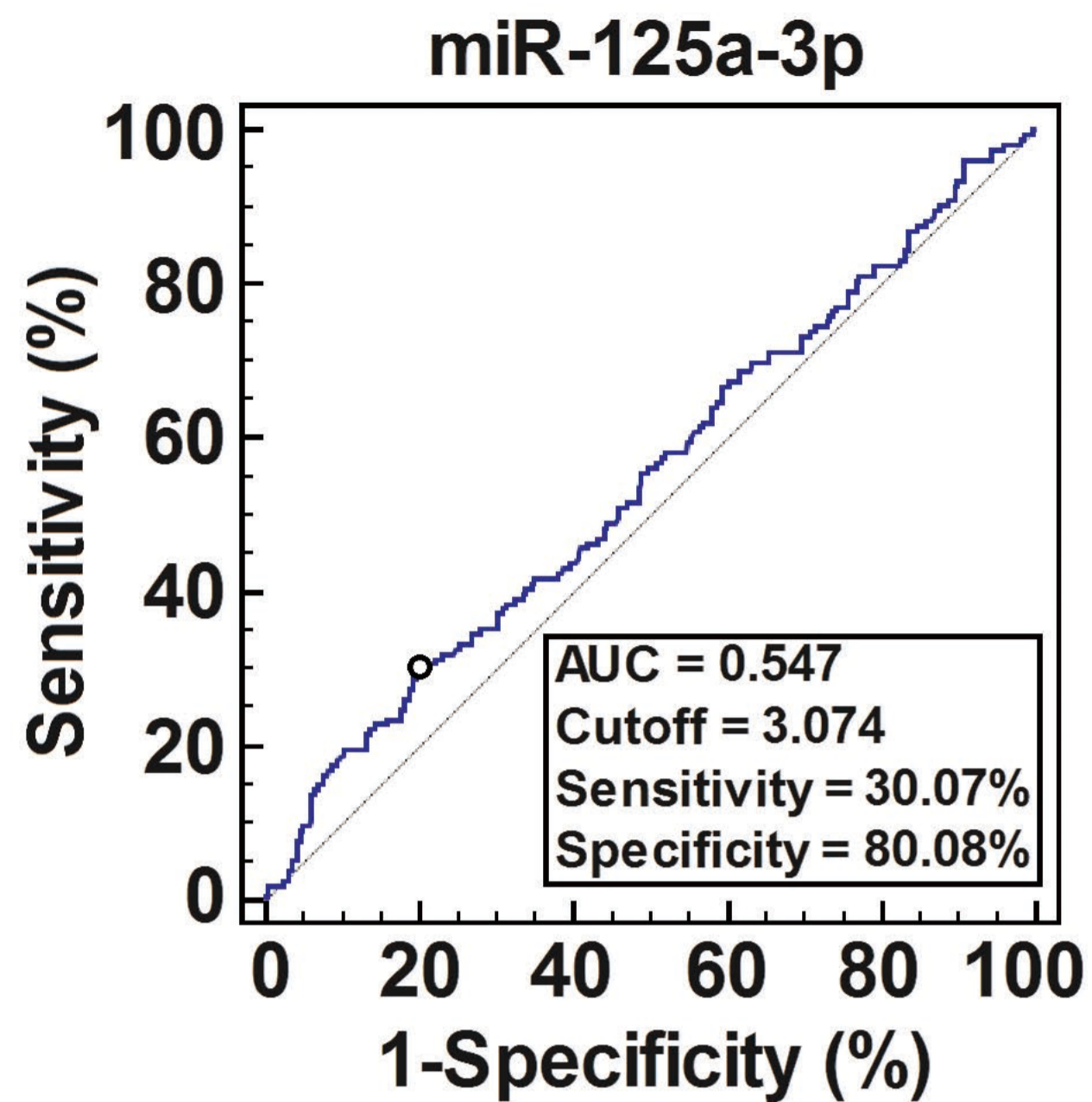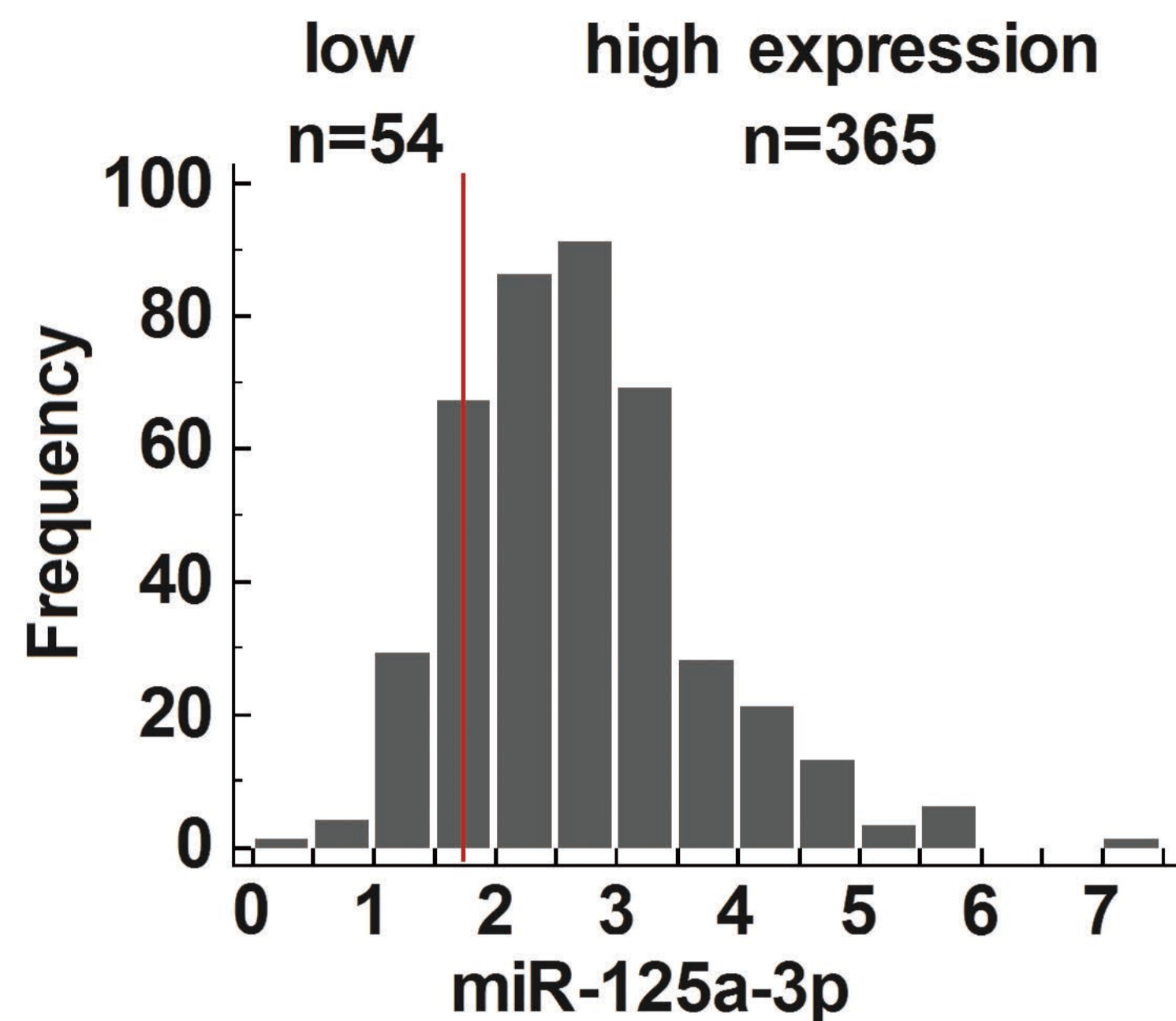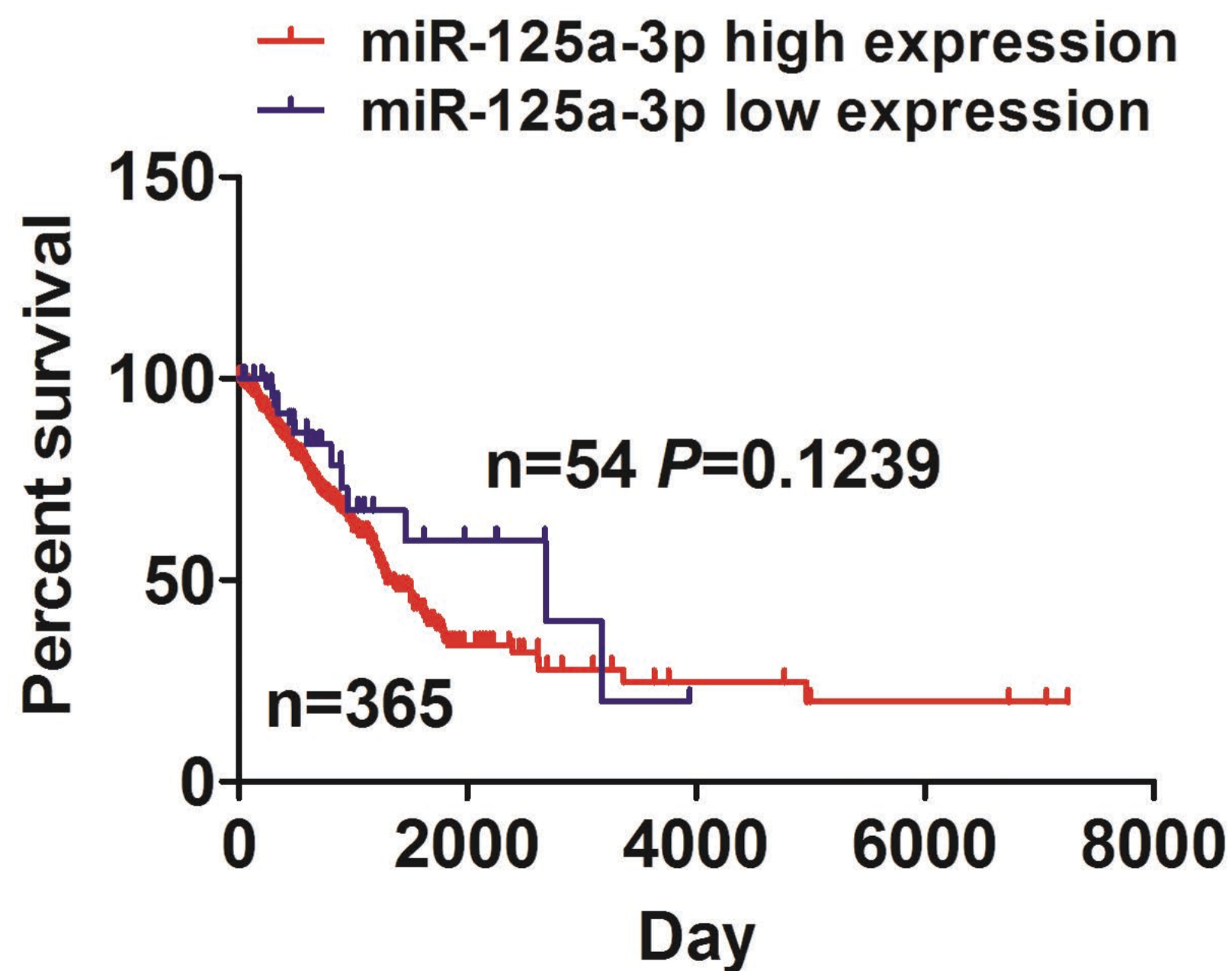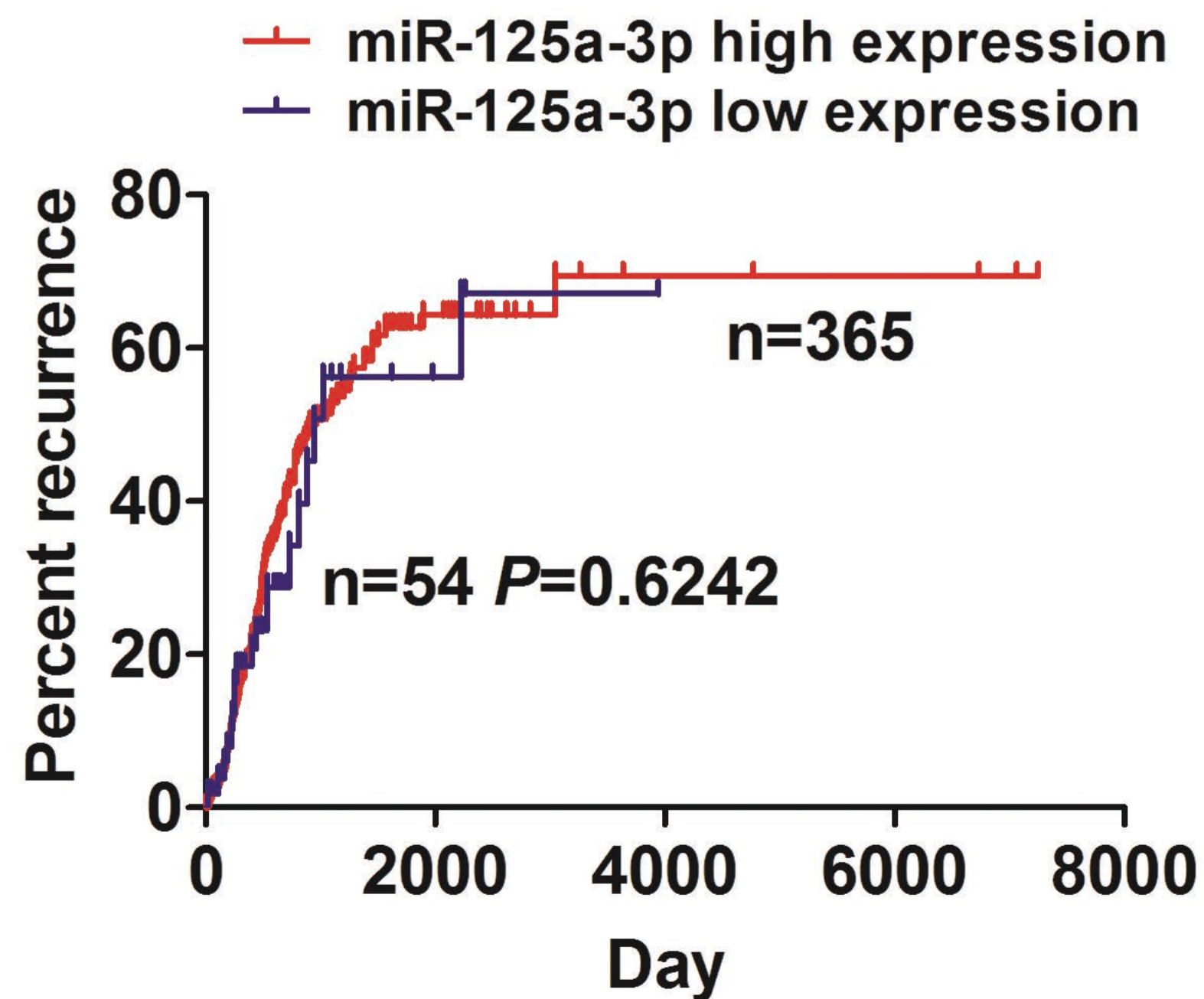

Supplement: Supplementary file 2 — Additional file 2: Figure S1. Kaplan-Meier analysis of the association of miR-125a-3pexpression with overall survival in patients with LUAC. [file 12935_2021_2241_MOESM2_ESM.pdf]

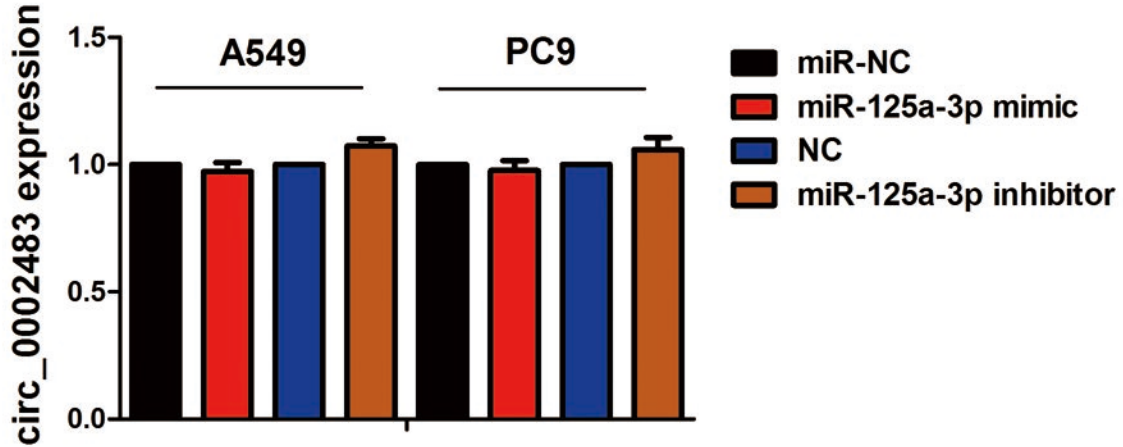

Supplement: Supplementary file 3 — Additional file 3: Figure S2. qRT-PCR analysis of the expression levels ofcirc_0002483 after transfection with miR-125a-3p mimics or inhibitors in A549and PC9 cells. [file 12935_2021_2241_MOESM3_ESM.pdf]

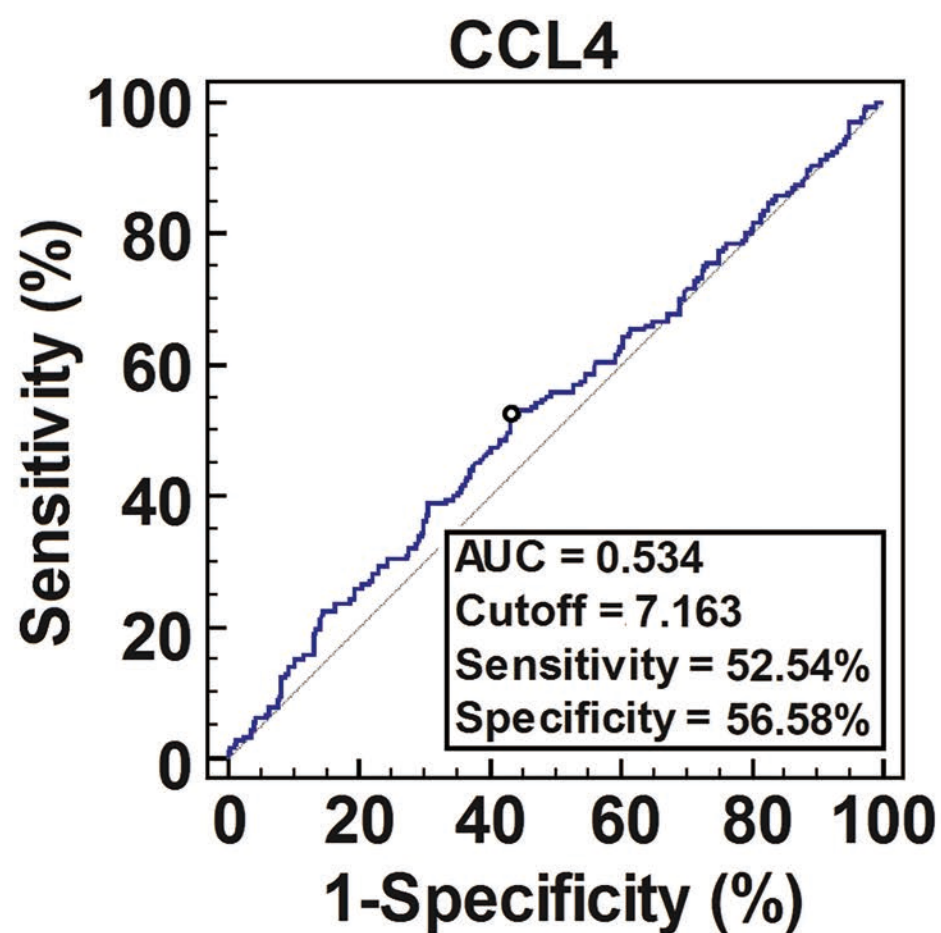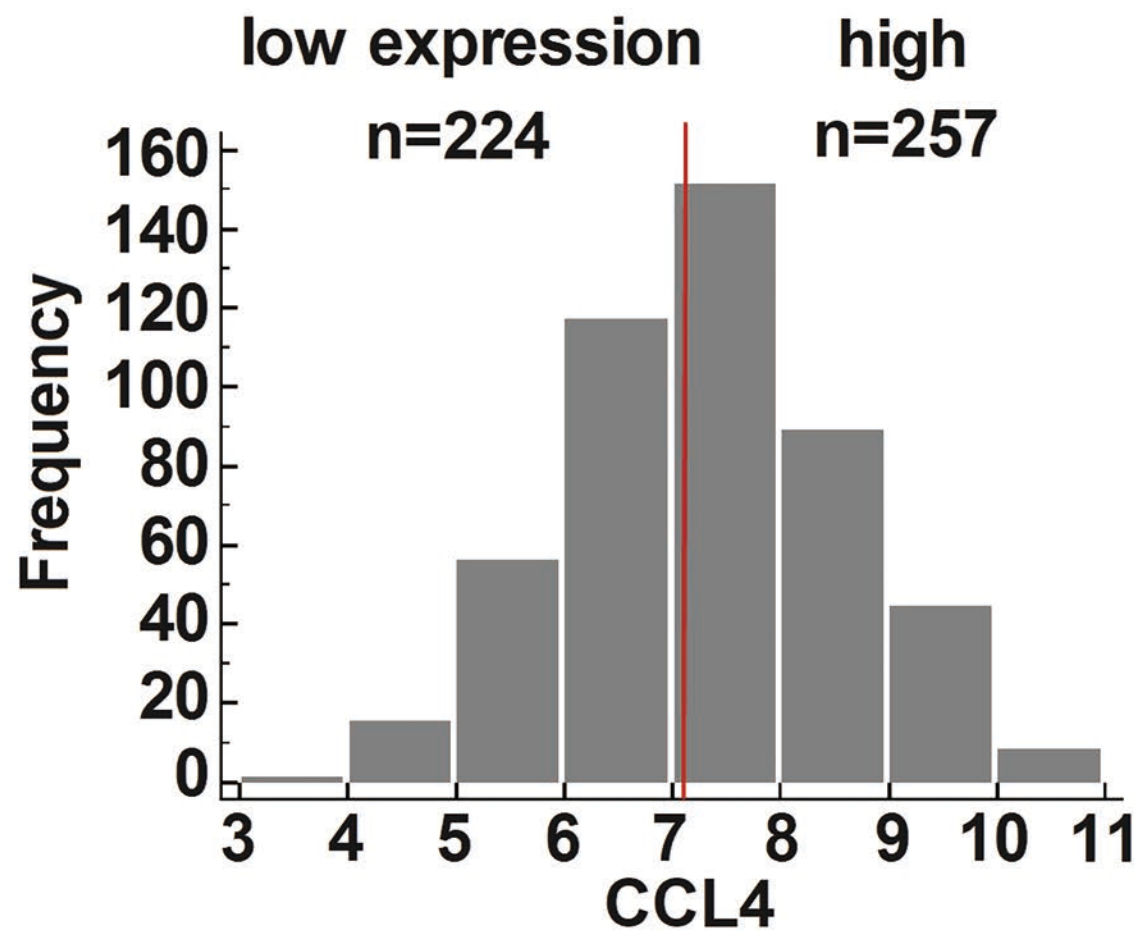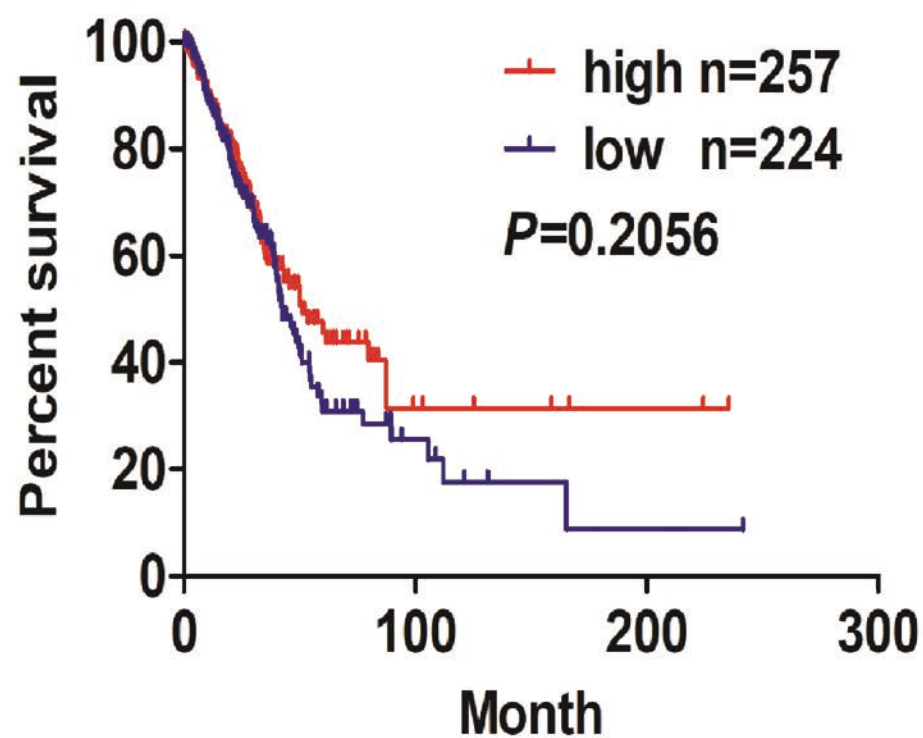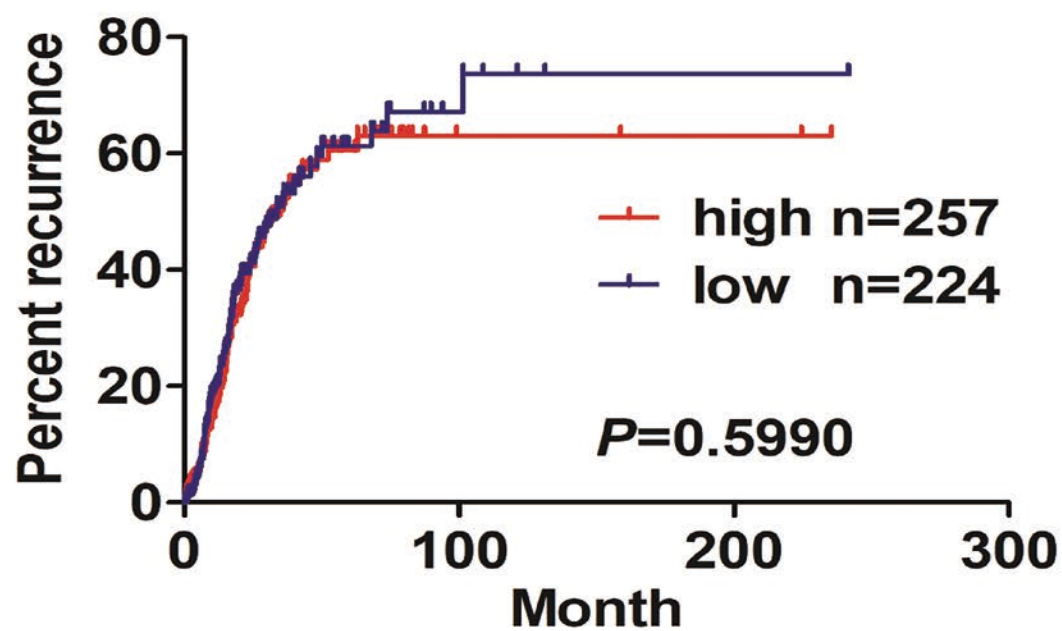

Supplement: Supplementary file 4 — Additional file 4: Figure S3. Kaplan-Meier analysis of the association of CCL4expression with overall survival in patients with LUAC. [file 12935_2021_2241_MOESM4_ESM.pdf]
